# Proof-of-Concept Study for an Enhanced Surrogate Marker of Endothelial Function in Diabetes

**DOI:** 10.1038/s41598-018-26931-2

**Published:** 2018-06-05

**Authors:** R. Dalan, S. Goh, Sun Bing, A. Seneviratna, C. T. Phua

**Affiliations:** 1 Tan Tock Seng Hospital, National Healthcare Group, Singapore, Singapore; 2 Lee Kong Chian School of Medicine, Nanyang Technological University, Singapore, Singapore; 3 Yong Loo Lin School of Medicine, Singapore, Singapore; 4 Nanyang Polytechnic, School of Engineering, Singapore, Singapore

## Abstract

Diabetes mellitus affects distal small vessels earlier and to a greater extent than proximal vessels. Vascular disease starts from activation of the endothelial cells, which if prolonged may lead to reduced distensibility of the vessel when maximally stimulated. Hence a device which measures distensibility of a distal vessel should be a good biomarker for subclinical disease. We have developed a device capable of measuring reactive hyperaemia induced changes in the radial artery flow, volumetric changes and accompanying effects on the vessel wall. The measurement is based on the magnetic flux disturbance upon haemodynamic modulation as blood flows through a uniformly applied magnetic field, and generates what we have termed the radial artery maximum distensibility index (RA-MDI). In a proof-of-concept study we found significant correlations between RA-MDI and cardiovascular risk factors, scoring systems and carotid artery intima-media thickness. Further large scale prospective studies need to be conducted to ascertain the correlations with cardiovascular events.

## Introduction

Patients with diabetes have accelerated atherosclerotic vascular disease with a higher disease burden^1^. Endothelial dysfunction is known to be initiated long before the formation of structural atherosclerotic changes in the arteries^2,3^. In patients with type 2 diabetes mellitus (T2DM), endothelial dysfunction is known to precede the development of hyperglycaemia and frank diabetes mellitus^4–6^. A landmark study in 1980 by Furchgott and Zawadzki demonstrated the obligatory role of the endothelium and endothelium-derived relaxing factor in modulating vascular tone^7^. Subsequently, a significant amount of research has led to the development of invasive and non-invasive tools to assess vasoreactivity in an attempt to identify individuals at risk for vascular disease and to examine the influence of treatment strategies. The underlying assumption of the research is that a sudden increase in shear stress can activate shear stretch receptors on the endothelial surface which trigger the release of vasodilatory molecules (nitric oxide and others) and cause subsequent arterial vasodilatation^8–10^. This endothelium-dependent vasodilation is impaired in type 1 and type 2 diabetes and in insulin resistance^11–13^.

Currently, there are several methods that measure endothelial function in a research setting such as cardiac catheterisation, venous occlusion plethysmography, ultrasound brachial artery flow-mediated dilation (FMD) and pulse amplitude tonometry (PAT)^9,10^.

Ultrasound brachial artery FMD is reported to be the least invasive method and provides a good indicator of endothelial function recognised in medical research. The concept that “tonically active flow-mediated dilatation occurs independent of changes in distending pressure in large peripheral artery in humans” was first demonstrated by Andersen and Mark in 1989^14^. Subsequently, the use of brachial artery FMD for measurement of endothelial dysfunction was described and a standardised protocol was developed^2^. Briefly, this method measures the flow-mediated changes in the diameter of the brachial artery using ultrasound. The brachial artery diameter is also affected by reflex stimuli resulting in direct sympathetic vasoconstriction^14^. It is a muscular artery and hence flow related dynamics will also be dependent to some extent on the muscular build of the individual. This method has high inter-user and intra-operator variability as a result of the angle and location of the ultrasonic scanner. It has very high initial set up costs and requires significant training for the operators^9,10^.

For PAT, the clinical instrument EndoPAT 2000 is commonly used to assess endothelial function through measurement of reactive hyperaemia in the microvascular system (i.e. capillaries). This method assesses the microvascular system to establish potential complications in the macrovascular system (e.g. arteries, veins). The microvascular system is reported to have substantial variability as a result of changes in environmental temperature and types of food intake^10^.

None of the above methods use the radial artery for the measurements. While the brachial artery lumen diameter is 3.6–4.4 mm, the radial artery (the smaller of the two terminal branches of the brachial artery) diameter is much smaller at 1.8–2.5 mm. Although the capillaries are much smaller and may provide an even earlier indicator, they are also prone to changes in the environment like exposure to cold.

Diabetes tends to affect the distal vessels more than the proximal vessels and the initial disease starts in the more peripheral smaller vessels. It is also very likely that subclinical atherosclerosis will manifest to a greater extent in the distal small vessels than the proximal vessels. In fact, it has been observed that an infusion of arginine vasopressin increases global forearm blood flow but causes a vasoconstriction in the radial arteries^15^. Basal and stimulated release of nitric oxide has been seen to result in radial artery vasodilatation and an increase in blood flow^16^.

We developed a device capable of measuring maximum distensibility in the radial artery using electromagnetic effects of haemodynamic modulation.

In contrast with brachial artery FMD, our device measures the electromagnetic changes that occur due to flow-mediated vasodilatation of the radial artery. It has been demonstrated that for brachial arteries, statistically significant vasodilation is only seen after 5 minutes of occlusion and although blood flow increases right after occlusion is reversed, vasodilation occurs only after 1 minute of release^17^. Hence, for measurement of brachial artery FMD, brachial artery diameter is measured at baseline and 1 minute after release of occlusion. Our algorithm is based on this principle but we use the maximum dilatation during the 5-minute period after the occlusion is released, since vasodilatation may in some occasions occur much earlier or later than the postulated 1 minute. We utilise an automated method of measurement and the algorithm allows us to adjust for heart rate and other variables. This results in potentially lower risk of inter- and intra-individual variations. This algorithm generates a final index which we have named as the radial artery maximum distensibility index (RA-MDI).

Endothelial vasodilator dysfunction in coronary and brachial arteries has been reported to be a predictive marker for cardiovascular events^18,19^. Currently, methods such as the Framingham risk scoring systems are commonly used to assess cardiovascular risk. Using our device, we measured the RA-MDI in 96 subjects (46 patients with T2DM and 50 controls) and studied correlations with common cardiovascular risk scores. We also assessed correlations with subclinical atherosclerosis using carotid artery intima-media thickness (CIMT) and traditional cardiovascular risk factors such as glycated haemoglobin (HbA1c), blood pressure (BP), lipid profile, and markers of inflammation.

## The process of measurement of the radial artery maximum distensibility index (RA-MDI)

**Figure 1:**
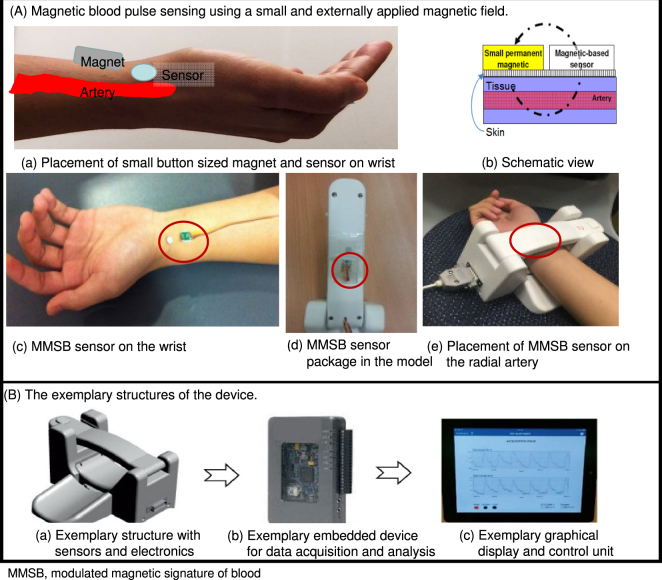
Magnetic blood pulse sensing and the exemplary structures of the device.

The developed device consists of two exemplary structured wrist straps with the modulated magnetic signature of blood (MMSB) sensor and magnet packaged on the top strap (Fig. 1A). The placement is such that it can be strapped on the wrist in close proximity to the radial artery (Fig. 1A). The sensors are connected to a receiver (exemplary embedded device for data acquisition and analysis) which wirelessly transfers the signals to an exemplary graphical display unit (Fig. 1B). The software programme (described later on) uses the algorithm to display the waveforms and calculate the final RA-MDI.

**Figure 2:**
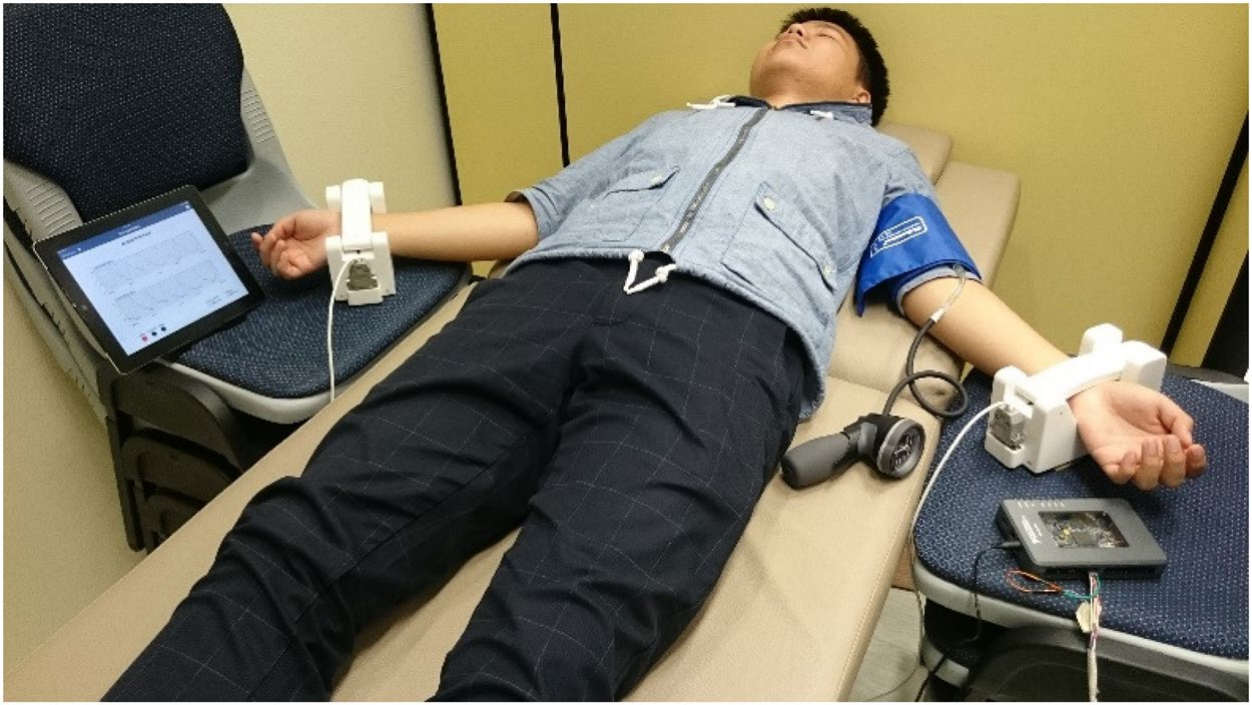
The process of obtaining the measurement.

**Figure 3:**
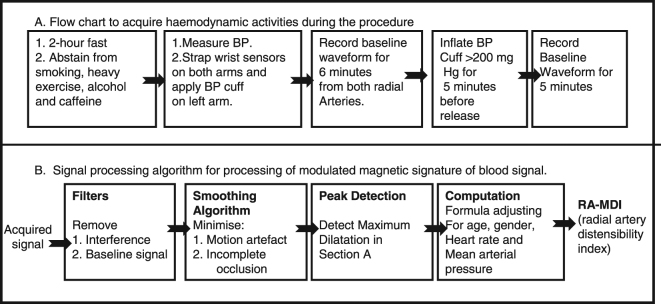
Flow chart and signal processing algorithm to acquire the haemodynamic activities during the procedure.

**Figure 4:**
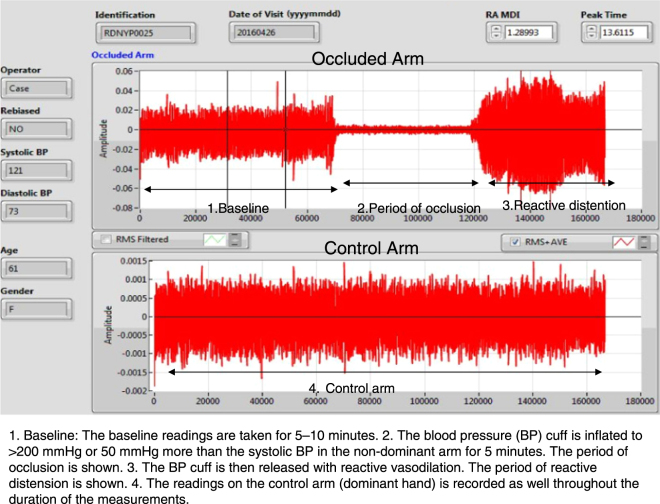
The final analysis page on the reader.

The measurements were undertaken in a hospital clinic room with a standardised room temperature of 25 °C. The procedure was as follows (Figs 2–4):

1. Subjects came for the study visit after an overnight fast (8 hours) and abstinence from smoking, alcohol and caffeine for 8 hours.
2. They were rested in a reclining position on a couch for 5–10 minutes (Fig. 2).
3. The baseline BP was measured using a standard sphygmomanometer in the dominant hand (i.e. right hand for right-handed individuals).
4. Subsequently, the BP cuff was applied on the non-dominant hand (i.e. left hand for right-handed individuals).
5. A wrist sensor strap (which contained the sensor and magnets to detect the difference in electromagnetic flux) was strapped on the subject’s right and left wrists.
6. Baseline waveforms were recorded for 5–6 minutes from both radial arteries.
7. The BP cuff was then inflated to >200 mmHg in the non-dominant hand and kept at that level for 5 minutes. During this time the waveforms from the radial artery were observed to document complete occlusion.
8. At exactly 5 minutes, the pressure was released and subsequent changes in the magnetic flux were measured for a total of 5 minutes.
9. An algorithm was used to calculate the RA-MDI adjusting for background noise, baseline variables, heart rate, and the measurements in the control arm.

## Clinical Validation

Initially we sought to determine intra-individual repeatability and reproducibility in healthy volunteers and in patients with T2DM. We measured the RA-MDI in 24 healthy volunteers with one operator. Each of the 24 individuals had the test administered on two consecutive days in similar conditions by the same operator. We performed a Bland-Altman analysis^20^ to assess the variability. The mean difference between the measurements was 0.05 (standard deviation, SD: 0.14) and all the measurements were within ±2 SD as seen in the Bland-Altman plot (Supplementary Fig. 7). Subsequently we performed the measurements on 10 T2DM subjects on two consecutive days. The mean difference between the measurements was within ±2 SD as seen in the Bland-Altman plot (Supplementary Fig. 8).

In the subsequent clinical correlation study, we examined the correlation between RA-MDI and cardiovascular risk. We recruited 50 healthy volunteers and 50 patients with T2DM. The inclusion criteria for T2DM group were individuals 21–80 years of age, diagnosed to have T2DM as per the World Health Organization criteria^21^, with normal renal function i.e. estimated glomerular filtration rate >60 mL/min/1.73 m^2^, and no history of previous atherosclerotic disease in any of the large vessels.

The control group consisted of healthy volunteers with no current diagnosis of diabetes mellitus or any other metabolic problems. Those with type 1 diabetes mellitus and intercurrent illness within the last 2 weeks were excluded.

Informed consent was obtained from all individuals and all aspects of the study were conducted in compliance with the principles of the Declaration of Helsinki. Approval was obtained from the institutional review board (National Healthcare Group, Domain Specific Review Board, and Ref: 2015/00424).

Demographic data and clinical parameters were collected, and standardised questionnaires were administered. BP was measured from the non-dominant hand in the supine position and the average of two readings was taken. All subjects were made to lie in a supine position for 10–15 minutes.

Carotid ultrasonography was performed using a 5.0–13.0 MHz multi-frequency high-resolution linear transducer probe (GE Logiq P5) by two operators trained before study initiation. Pilot examination on 23 volunteers showed acceptable limits of inter- and intra-user agreement with a coefficient of variance of ±0.1 by Bland-Altman analysis. The Auto-IMT^TM^ software was used for CIMT measurements in order to optimise reproducibility. These measurements were made following the recommendations of the Mannheim CIMT consensus^22^. Subsequently they underwent the RA-MDI procedure described previously.

Blood sampling was done for HbA1c, fasting glucose, lipid panel, and highly sensitive C-reactive protein (hsCRP). HbA1c was measured by immunoturbidimetric assay using the Beckman Coulter Synchron LX®20 (Beckman Coulter Inc., Brea, CA, USA) clinical chemistry analyser. Lipids were measured using standard coupled enzymatic methods. Low-density lipoprotein cholesterol (LDL-C) was calculated by the Friedewald equation. hsCRP was measured by turbidimetry.

Cardiovascular risk scores were calculated using the Framingham risk scoring system found at http://www.framinghamheartstudy.org/risk-functions/cardiovascular-disease/index.php based on the Framingham Heart Study (FHS)^23^. The scores for 10-year (FHS10) full cardiovascular disease (CVD) risk were calculated. Subsequently, 30-year (FHS30) hard CVD and full CVD risk scores were calculated using both the lipids and body mass index (BMI) algorithms. The following cut-offs were used to risk stratify the subjects into low, moderate and high risk groups. For FHS10, <10%: low risk, 10–20%: moderate risk, and ≥20%: high risk^23^. For FHS30 (lipids and BMI), ≥40% was categorised as high risk^24^. Based on the United Kingdom Prospective Diabetes Study (UKPDS), the UKPDS risk score was also calculated. The UKPDS Risk Engine is a definitive model for predicting absolute risk of CVD in male and female patients with T2DM. It provides risk estimates and 95% confidence intervals in patients with T2DM not known to have heart disease. These can be calculated for any given duration of T2DM based on current age, sex, ethnicity, smoking status, systolic BP, presence or absence of atrial fibrillation, and levels of HbA1c, total cholesterol and high-density lipoprotein (HDL) cholesterol^25^. We also calculated the Action in Diabetes and Vascular Disease: Preterax and Diamicron-MR Controlled Evaluation (ADVANCE) risk scores^26,27^, another score developed especially for diabetes patients and has been seen to outperform the FHS when used in patients with diabetes to estimate cardiovascular risk.

The datasets generated during and/or analysed during the current study are available from the corresponding author on reasonable request.

## Statistical Analysis

The planned sample size of 50 patients with T2DM (and 50 controls) was based on R-square values from (univariate or multivariate) linear regression and the following assumptions: desired R-square of 0.8, null R-square of 0.6 (i.e. R-square values below 0.6 were undesirable), 5% type I error rate, 80% power, and up to 10 predictors in the linear regression model.

Statistical analyses were performed using Stata (version 13.1, StataCorp LP, College Station, TX). Significance tests were two-sided at the 5% significance level. Results are summarised for the overall cohort and stratified by T2DM status. Results are reported as mean, standard deviation (SD) or median, interquartile range (IQR), and range (minimum–maximum) for continuous variables, and count (n) and percentage for categorical variables. Mann-Whitney U^28^ test was used to compare the RA-MDI between the T2DM and control groups. Correlation between RA-MDI and cardiovascular risk scores, traditional cardiovascular risk factors and vascular biomarkers was assessed using the Spearman rank-order method^29^. Simple and multiple linear regressions were used to evaluate the relationship between RA-MDI and the measures of cardiovascular risk. Ability of the RA-MDI to differentiate between high and low cardiovascular risks using the Framingham risk scores FHS10 Lipids and BMI, and FHS30 Lipids and BMI (full CVD outcomes) was assessed using receiver operating characteristic (ROC) curves^30^.

## Results

The baseline characteristics are described in Table 1. Briefly, 96 subjects were enrolled (46 with diabetes and 50 healthy volunteers. The healthy volunteers were younger compared to patients with T2DM. Most of the subjects recruited belonged to the Chinese ethnic group. Patients with T2DM had a higher BMI, waist circumference, and systolic and diastolic BP compared to healthy volunteers. Patients with T2DM had a better lipid profile in terms of LDL cholesterol and non-HDL cholesterol. This is probably because all diabetes patients were on treatment with statins. As expected, the patients with T2DM had a much higher cardiovascular risk estimated by all the Framingham risk scores. Amongst the healthy volunteers, using the standard cut-offs for FHS10 (Lipids) and FHS30 (Lipids)-full CVD risk, all subjects were in the low risk category. Using the FHS10 (BMI) and FHS30 (BMI)-full CVD risk cut-offs, one individual was at moderate–high risk. Amongst the patients with T2DM, for FHS10 (Lipids), six (13%) were classified as low risk, 21 (46%) as moderate risk and 19 (41%) as high risk. For FHS10 (BMI), five (11%) were classified as low risk, 14 (30%) as moderate risk and 27 (59%) as high risk. Estimating risk in T2DM patients using FHS30 (Lipids)-full CVD and FHS30 (BMI)-full CVD showed that 41 (89%) and 44 (96%) were at high risk respectively.

**Table 1:** Baseline characteristics.

	All	Healthy volunteers	Type 2 diabetes mellitus
Total number, n	96	50	46
Age, Median (IQR), Range	40.5 (28), 22–72	27.5 (9), 22–55	55 (15), 30–69
Male, n (%)	41 (42.7)	17 (34)	24 (52.2)
Ethnicity, n (%)			
Chinese	67 (69.8)	39 (78)	28 (60.9)
Malay	17 (17.7)	10 (20)	7 (15.2)
Indian	12 (12.5)	1 (2)	11 (23.9)
Duration of diabetes, years, Median (IQR), Range	11 (12), 1–35	NA	11 (12), 1–35
BMI, kg/m^2^, Median (IQR), Range	25.4 (7.3), 17.4–43.3	21.6 (6.8), 17.6–43.3	26.3 (4.5), 17.4–38.6
Waist circumference, cm, Mean (SD)	88.7 (12.4)	84.8 (12.1)	92.2 (11.9)
Systolic BP, mmHg, Mean (SD)	124.3 (15.5)	115.5 (11.3)	133.5 (14.5)
Diastolic BP, mmHg, Mean (SD)	71.4 (10.1)	67.4 (9.1)	75.7 (9.6)
HbA1c, (%) Median (IQR), Range	6.4 (2.4), 5–13.1	5.4 (0.3), 5–6.4	7.8 (2.0), 6.2–13.1
Non-HDL cholesterol, mmol/L, Mean (SD)	3.4 (1.0)	3.5 (0.9)	3.3 (1.0)
LDL cholesterol, mmol/L, Mean (SD)	2.9 (0.9)	3.1 (0.8)	2.5 (0.9)
hsCRP, mg/L, Median (IQR), Range	0.9 (2.2), 0.2–9.0	0.4 (1.05), 0.2–3.5	1.5 (2.9), 0.2–9.0
Average CIMT, mm Median (IQR), Range	0.5 (0.2), 0.4–1.0	0.5 (0.1), 0.4–0.6	0.7 (0.2), 0.5–1.0
Framingham Risk Scoring (%)			
FHS10 (Lipids) Median (IQR), Range	3.7 (16.9), 0.2–30	0.9 (0.8), 0.2–7.5	18.1 (14.4), 3–30
FHS10 (BMI) Median (IQR), Range	3.3 (22.1), 0.2–30	0.9 (1), 0.2–13.7	24.15 (15.3), 1.9–30
FHS30 (Lipids)-full CVD, Median (IQR), Range	19 (58.5), 1–87	6 (5), 1–28	65.5 (19), 16–87
FHS30 (Lipids)-hard CVD, Median (IQR), Range	10.5 (43.5), 0–81	3 (2), 0–16	46 (21), 8–81
FHS30 (BMI)-full CVD, Median (IQR), Range	17.5 (67.5), 1–89	5 (5), 1–44	73 (18), 11–89
FHS30 (BMI)-hard CVD, Median (IQR), Range	9.5 (55.5), 0–84	2 (3), 0–30	58 (23), 6–84
ADVANCE score, %, Median (IQR), Range	3 (4), 0–13	NA	3 (4), 0–13
UKPDS score, %, Median (IQR), Range	11 (28), 1–52	NA	11 (28), 1–52
RA-MDI			
Range	0.03–2.72	0.03–0.81	0.22–2.72
Mean (SD)*	0.6 (0.49)	0.31 (0.16)	0.92 (0.53)
Median (IQR)**	0.42 (0.5)	0.28 (0.13)	0.76 (0.46)

ADVANCE, Action in Diabetes and Vascular Disease: Preterax and Diamicron-MR Controlled Evaluation; BP, blood pressure; CIMT, carotid artery intima-media thickness; CVD, cardiovascular disease; FHS10, Framingham risk scoring for 10 years; FHS30, Framingham risk scoring 30 years; HbA1c: glycated haemoglobin; hsCRP, high sensitivity C-reactive protein; IQR, interquartile range; Non-HDL, non-high density lipoprotein; LDL, low-density lipoprotein; RA-MDI, radial artery maximum distensibility index; SD, standard deviation; UKPDS: United Kingdom Prospective Diabetes Study.

*Two-sample t-test p-value < 0.0001, **Mann-Whitney test p-value < 0.0001.

The RA-MDI scores were significantly higher in T2DM (Median 0.76 (IQR: 0.46)) compared to healthy volunteers (Median: 0.28 (IQR: 0.13), p < 0.001).

Spearman correlation analyses showed strong correlations between the RA-MDI scores and all the scoring systems: FHS (10 and 30 years): r_s_ = 0.7, p < 0.001; UKPDS: r_s_ = 0.7, p < 0.001, and ADVANCE: r_s_ = 0.7, p < 0.001 (Table 2). Significant correlations were also seen with HbA1c (r_s_ = 0.6, p < 0.001) and with average CIMT (r_s_ = 0.6, p < 0.001). Linear regression analysis also showed that RA-MDI correlated significantly with all the risk scoring systems and CIMT, p < 0.001 (Table 3). Significant correlations with the traditional cardiovascular risk factors, HbA1c (p < 0.001), age (p < 0.001), systolic and diastolic BP (p < 0.001), and BMI (p = 0.01), were also observed.

**Table 2:** Spearman correlations between RA-MDI scores and the risk scoring systems and CIMT.

	Spearman correlation (r_s_)	P value
FHS10 (Lipids)	0.7059	≤0.001
FHS10 (BMI)	0.7242	≤0.001
FHS30 (Lipids)-full CVD	0.6889	≤0.001
FHS30 (Lipids)-hard CVD	0.7023	≤0.001
FHS30 (BMI)-full CVD	0.7083	≤0.001
FHS30 (BMI)-hard CVD	0.7281	≤0.001
ADVANCE	0.6884	≤0.001
UKPDS	0.7088	≤0.001
HbA1c	0.5778	≤0.001
Average CIMT	0.5900	≤0.001

ADVANCE, Action in Diabetes and Vascular Disease: Preterax and Diamicron-MR Controlled Evaluation; CIMT, carotid artery intima-media thickness; CVD, cardiovascular disease; FHS10, Framingham risk scoring for 10 years; FHS30, Framingham risk scoring 30 years; HbA1c, glycated haemoglobin; RA-MDI, radial artery maximum distensibility index; UKPDS, United Kingdom Prospective Diabetes Study.

P value < 0.01 is considered significant.

**Table 3:** Linear regression analysis showing the association of cardiovascular risk scores and cardiovascular risk factors with RA-MDI.

	Correlation coefficient	95% confidence interval		P value
FHS10 (Lipids)	0.0292	0.0196	0.0387	<0.001*
FHS10 (BMI)	0.0270	0.0187	0.0354	<0.001*
FHS30 (Lipids)-full CVD	0.0102	0.0073	0.0131	<0.001*
FHS30 (Lipids)-hard CVD	0.0124	0.0082	0.0166	<0.001*
FHS30 (BMI)-full CVD	0.0097	0.0071	0.0123	<0.001*
FHS30 (BMI)-hard CVD	0.0114	0.0081	0.0146	<0.001*
ADVANCE	9.6679	4.6630	14.6728	<0.001*
UKPDS	1.6350	0.8316	2.4383	<0.001*
Average CIMT	1.6544	0.9372	2.3716	<0.001*
Maximum CIMT	0.9675	0.6110	1.3239	<0.001*
C-reactive protein	0.0334	−0.0097	0.0765	0.128
Age	0.0204	0.0145	0.0264	<0.001*
Gender				
Male				Ref
Female	−0.2630	−0.4741	−0.0519	0.015*
Ethnicity				
Chinese				Ref
Malay	0.0454	−0.2604	0.3511	0.769
Indian	0.3856	0.0662	0.7051	0.019
Body mass index	0.0252	0.0058	0.0446	0.012*
Systolic blood pressure	0.0118	0.0067	0.0170	<0.001*
Diastolic blood pressure	0.0144	0.0057	0.0232	0.001*
Non-HDL cholesterol	−0.0762	−0.1778	0.0254	0.140
LDL cholesterol	−0.1074	−0.1954	−0.0194	0.017
HbA1c	0.1049	0.0611	0.1486	<0.001*
Subject type				
Control				Ref
Diabetes mellitus	0.6174	0.4562	0.7786	<0.001*

ADVANCE, Action in Diabetes and Vascular Disease: Preterax and Diamicron-MR Controlled Evaluation; CIMT, carotid artery intima-media thickness; CVD, cardiovascular disease; FHS10, Framingham risk scoring for 10 years; FHS30, Framingham risk scoring 30 years; HbA1c: glycated haemoglobin; non-HDL, non-high density lipoprotein; LDL, low-density lipoprotein; UKPDS: United Kingdom Prospective Diabetes Study.

P value < 0.01 is considered significant.

**Figure 5:**
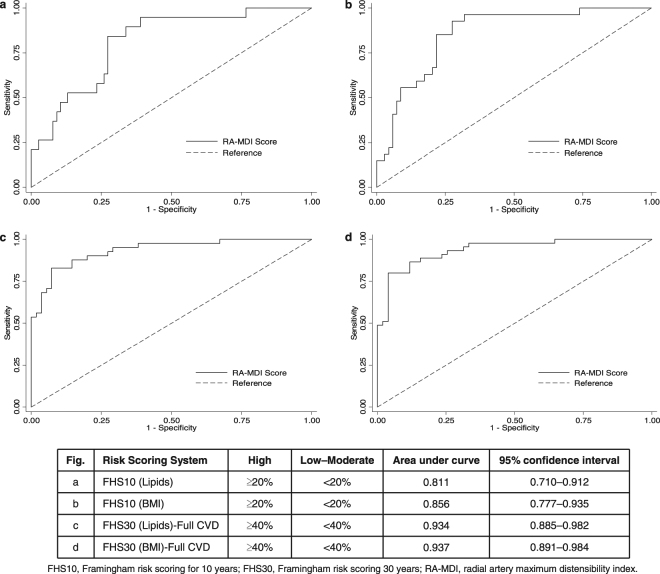
Receiver operating curves for RA-MDI score used for identifying high cardiovascular risk.

In the overall population, RA-MDI was able to differentiate high cardiovascular risk (FHS10 Lipids) with an area under the curve (AUC) of 0.81 (95% CI: 0.71–0.91) (Fig. 5) using an RA-MDI cut-off of ≥0.653. Using a lower cut-off of 0.641 for RA-MDI, it was able to differentiate high cardiovascular risk for other risk engines: FHS10 (BMI) (AUC: 0.86 (95% CI: 0.78–0.94)), FHS30 (Lipids)-full CVD risk (AUC: 0.93 (95% CI: 0.89-0.98)) and FHS30 (BMI)-full CVD (AUC: 0.94 (95% CI: 0.89-0.98)) (Fig. 5).

## Discussion

Our device is based on the same principles as the traditional brachial artery FMD wherein brachial artery diameter is measured during three conditions, at baseline (after about 10 minutes of supine rest) and during reactive hyperaemia (induced by inflation to 250 mmHg and then deflation of a sphygmomanometer cuff around the forearm)^8^. In a proof-of-concept study, it has been seen that the radial artery diameter can be increased noninvasively via a reactive hyperaemia protocol^31^. Instead of radial artery diameter measurements, we measured the magnetic modulation of blood which is affected by shear stress and we quantitatively measured the changes after a period of occlusion.

We chose the radial artery to obtain these measurements because it is easily accessible clinically and causes minimum discomfort to the patient. In a cadaveric study using histopathology to accurately grade atherosclerosis, a significant correlation was observed between the pathology in the radial artery and the pathologic left coronary artery and bifurcation of the carotid arteries^32^. Moreover, radial artery intima-media thickness has been associated with coronary artery disease^33^. In one study looking at the arterial wall elasticity of the radial artery, the elasticity correlated with microvascular complications of diabetes (like retinopathy and nephropathy) in T2DM patients^34^. A frequency based domain-optical coherence tomography study has also demonstrated correlations of radial artery intima-media ratio with coronary thin cap fibroatheroma^35^.

We found significant differences between patients with T2DM and healthy volunteers in our study. Diabetes is known to be a significant cardiovascular risk factor and significant differences are seen in other endothelial function measurements as well^36,37^, with these measurements correlating to endothelial dysfunction. We found significant correlations of diabetes with cardiovascular risk factors such as age, gender, BMI, and systolic and diastolic BP. Similar correlations have been seen for brachial artery FMD in a large study comprising 2,511 Chinese individuals^38^. We saw a significant correlation with CIMT in this study. The brachial artery FMD has not been seen to associate with CIMT in middle-aged and elderly individuals^39,40^. It has been postulated that brachial artery FMD may be distinct and independent stages in the complex atherosclerotic process. However since the radial artery intima-media thickness has been associated with cardiovascular risk and elasticity has been associated with vascular function, measurement of reactive hyperaemia-induced maximum possible dilatation, may also reflect the atherosclerotic process occurring in the intima-media of the radial artery. Radial artery intima-media thickness has also been shown to regress or decrease after percutaneous intervention in patients with acute coronary syndrome demonstrating early reversibility when compared to the carotid artery^41^. However this correlation needs to be confirmed in larger population-based studies.

We found significant correlations of the RA-MDI with the Framingham risk scores as has been seen with the brachial artery FMD^42,43^. There were also significant correlations with all the cardiovascular risk engines in this study.

The main advantage of our method over current methods is its user-friendliness and minimum inter-individual differences between measurements. Specialised training for individuals performing the measurement is not required. The device does not require use of any disposable probes making it very cost effective. There are no expensive set-up processes needed unlike ultrasound-based measuring devices. Finally, our device demonstrates potential ability to be used for longitudinal follow up.

The main limitations of the current study is that we have done these estimations only in a small sample size as a proof of concept and this device will need validation in a large consecutive patient cohort. Further clinical utility of the device could be examined by correlating with major adverse cardiac and cerebrovascular events and assessing its ability to risk-stratify patients. Reversibility in the measurements with therapeutics also needs to be assessed in further studies.

## Conclusion

We have developed for the first time a device measuring reactive hyperaemia-induced maximum possible distension in the radial artery. Our pilot study has demonstrated proof of concept that our device correlates with measures of traditional cardiovascular risk factors and risk engines. Larger population-based studies are needed to validate these correlations and evaluate them with strong endpoints like cardiovascular events.

## Methods

### Development of the algorithm

The concept of magnetic blood pulse measurement^44^ revolves around the application of a uniform magnetic field in the vicinity of the major artery. At the location where the applied magnetic field is placed, the pulsatile nature of blood flow will result in volumetric changes of blood that will disturb the magnetic field. This unique magnetic disturbance is dependent on the volumetric changes in blood and can be acquired using a giant magnetoresistance-based magnetic sensor operating at room temperature. This principle is termed the MMSB. The volumetric changes in blood flow after an occlusion will result in variation in magnetic flux disturbance that is quantifiable and is hypothesised to be able to quantify endothelial dysfunction for assessment of macro-CVD associated with diabetes mellitus.

The proposed hypothesis is based on theoretical findings where a correlation study was performed successfully employing a laser Doppler in concurrence with the magnetic flux disturbance method, using the time measured from maximum occlusion to maximum blood flow^45^. The key method used to acquire such haemodynamic characteristics is based on the measurement of magnetic flux disturbance as blood flows through a uniformly applied magnetic field as shown in Fig. 1.

The MMSB sensing method was developed into a device as shown in Fig. 1. The method for data acquisition is outlined under the process of measurement (Fig. 3A) and a typical waveform is acquired from these (Fig. 4).

Using the waveform acquired, a signal processing algorithm is developed to assess the changes in the blood haemodynamics before and after an occlusion as shown in Fig. 3B. The AC power supply in Singapore operates at 240 V/50 Hz. Thus, the key noise contributions for the design come from the 50 Hz frequency of the AC power supply. We designed the filtering solutions for our device as shown in Supplementary Fig. 1.

The first stage filtering removes the 50 Hz noise using a bandstop filter as illustrated in Fig. 1. The default value of A_b_ (where A_b_ denotes a level on the magnitude squared response of the filter in decibels) is −3 dB as the filter is designed to remove 50% of the noise power within the stopband. An infinite impulse response (IIR) notch filter was designed, using the LabVIEW Digital Filter Design Toolkit, with a 50 Hz centre frequency and a stopband frequency of 5 Hz as illustrated in Supplementary Fig. 2. In Supplementary Fig. 2b, *f*_0_ denotes the centre frequency and Δ*f* denotes the frequency bandwidth at *A*_*b*_. The transfer function for the notch filter is shown in Equation 1.

1 $$H(z)=\frac{{b}_{0}(1+\frac{{b}_{1}}{{b}_{0}}{z}^{-1}+{z}^{-2})}{1+{a}_{o}{z}^{-1}+{a}_{1}{z}^{-2}}$$

where *a*_0_, *a*_1_, *b*_0_, and *b*_1_ are filter coefficients.

The filter coefficients are defined in Table 4.

**Table 4:** The filter coefficients (a_0_, a_1_, b_0_, b_1_).

Coefficients	Value
a_0_	0.0302984
a_1_	0.9229900
b_0_	0.9614950
b_1_	0.0302984

The second filtering stage involves removing high frequency interference using a finite impulse response (FIR) low pass filter designed to further remove noise greater than 30 Hz. The cut-off frequency of the filter was chosen at 30 Hz as it is at least 5 times lower than the sampling frequency which is at 200 Hz, and about 2.5 times lower than the Nyquist limit. With this setting, the dynamic range of response of the cardiovascular vessels due to vasodilation during measurement can be fully captured. The final filtering stage removes the baseline signal by subtracting the DC value, determined using the LabVIEW Basic DC/RMS VI block, from the signal (Supplementary Fig. 3).

Removal of motion artefact and incomplete occlusion is achieved in two stages. First, the root mean square value of each stage is determined. This value is used to determine whether a signal falls within the valid range. Second, an additional tier of smoothing is achieved through further averaging of the signal (Supplementary Fig. 4). Peak detection of waveform trend on the occluded arm is done using the LabVIEW Array Max and Min VI (Supplementary Fig. 5). Heart rate is determined by counting the number of peaks per minute (Supplementary Fig. 6).

The signal processing is developed based on the fact that the volumetric changes in blood flow are a direct correlation to the area under the curve. In addition, parameters to discern muscular physical differences between genders, mean arterial BP and age are also included to account for the difference in baseline for each person. We observed that each individual arrives at the measurement site with varying states of mind and physical stress (e.g. relaxed, panting, stressed, etc.). In addition, throughout the process of data acquisition, each has a different state of mind (e.g. sleeping, thinking of work, shocked by environmental noise, etc.). Therefore, in order to account for these differences, average heart rate of the individual during resting, occlusion and relaxation phases of measurement were calculated and included into the final algorithm to compensate for the differences.

The algorithm generates a final index, which we have named the RA-MDI, which was used for subsequent correlation studies with blood markers and other cardiovascular markers. The final algorithm was implemented into the deployable platform myRIO from National Instruments (Fig. 1B). The waveform generated is seen in Fig. 4 (also see Additional Information).

### Data Availability

Detailed data regarding this manuscript is available upon request to the corresponding author.

## Electronic supplementary material

Supplementary Figures

The process of measurement of the RA-MDI
