# Supplementary material for: Proof-of-Concept Study for an Enhanced Surrogate Marker of Endothelial Function in Diabetes
Source: Sci Rep. 2018 Jun 5;8:8649. doi: 10.1038/s41598-018-26931-2 (PMC5988679; doi:10.1038/s41598-018-26931-2)
Supplement: Supplementary file 1 — Supplementary Figures [file 41598_2018_26931_MOESM1_ESM.pdf]

Proof-of-Concept Study for an Enhanced Surrogate Marker of Endothelial Function in Diabetes

*Dalan R, Goh S, Sun Bing, Seneviratna A, Phua CT

Supplementary Figures Legends

Supplementary Figure 1 Design of the filtering solutions.

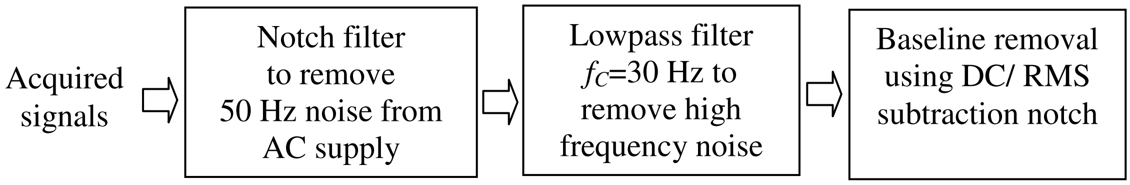

Supplementary Figure 2 Implementation of notch filter in LabVIEW.

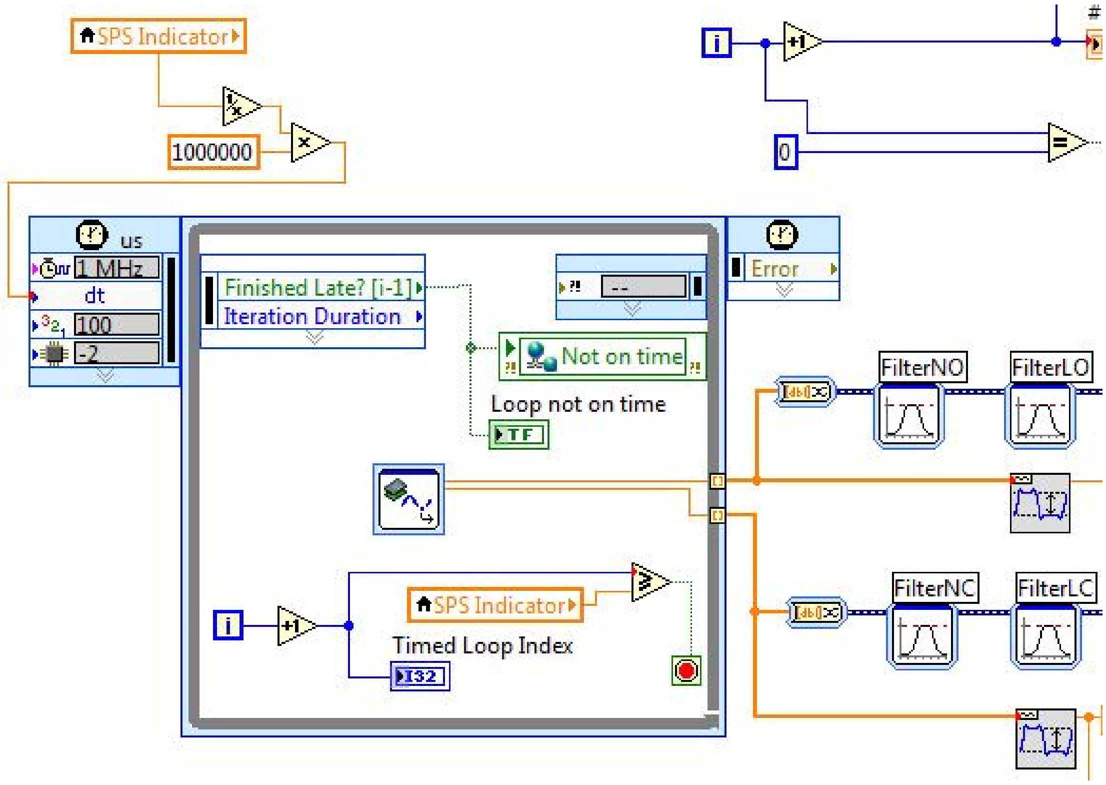

(a) Overview of the implementation of the notch filter

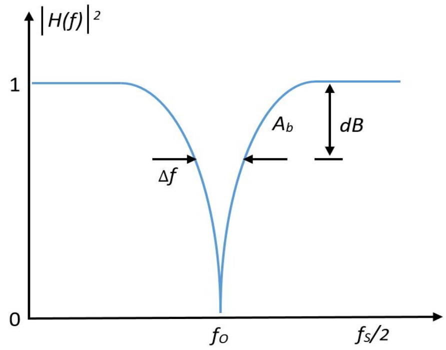

(b) Typical response of notch filter

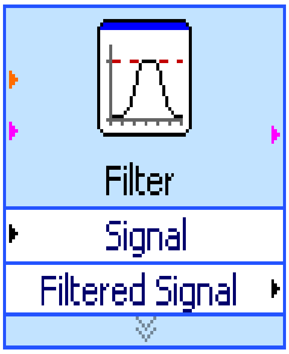

(c) LabVIEW filter block

Supplementary Figure 3 The final filtering stage which removes the baseline signal by subtracting the DC value determined using the LabVIEW Basic DC/RMS V1 block from the signal.

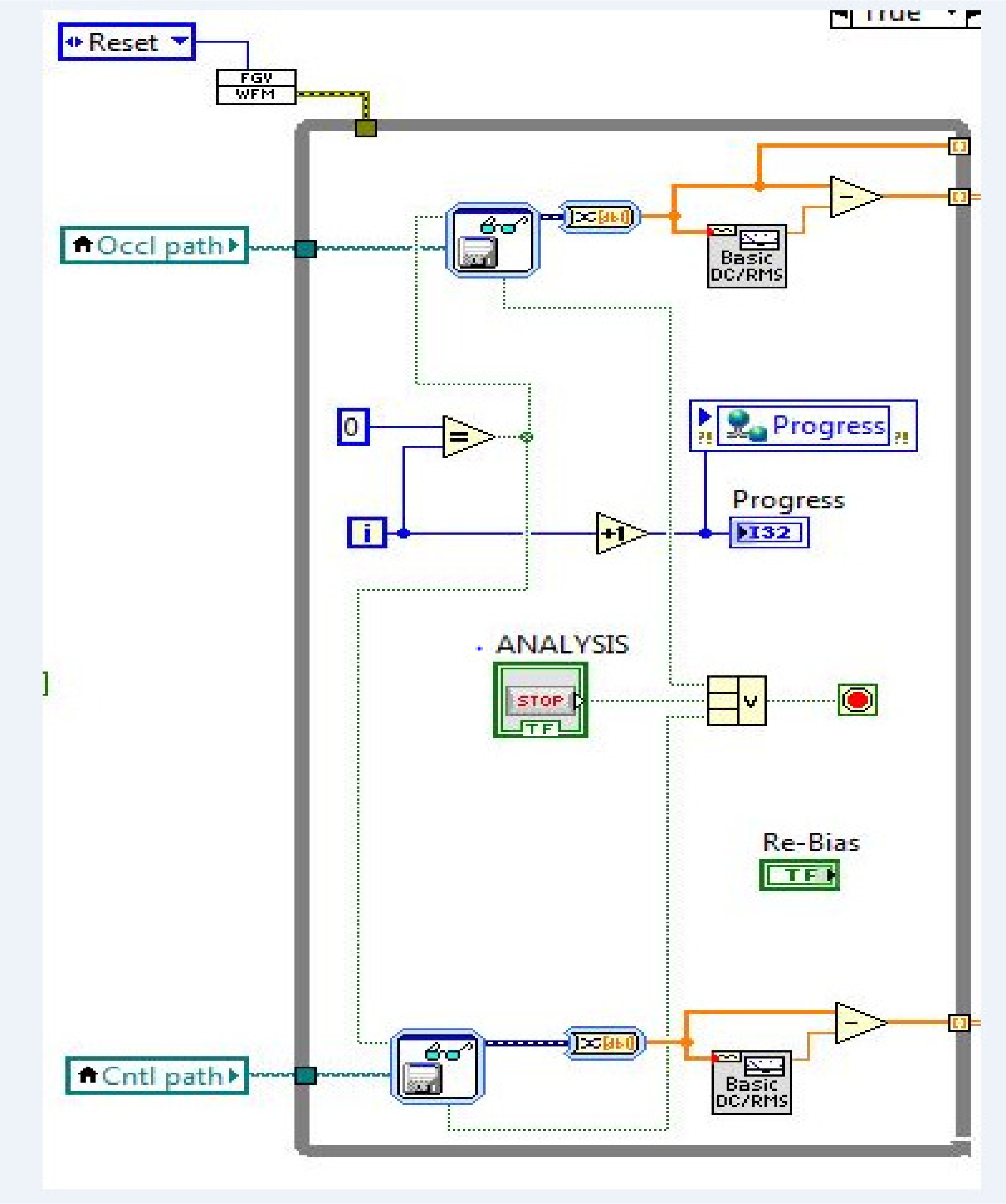

Supplementary Figure 4 The smoothing algorithm achieved through removal of motion artefact and averaging of the signal.

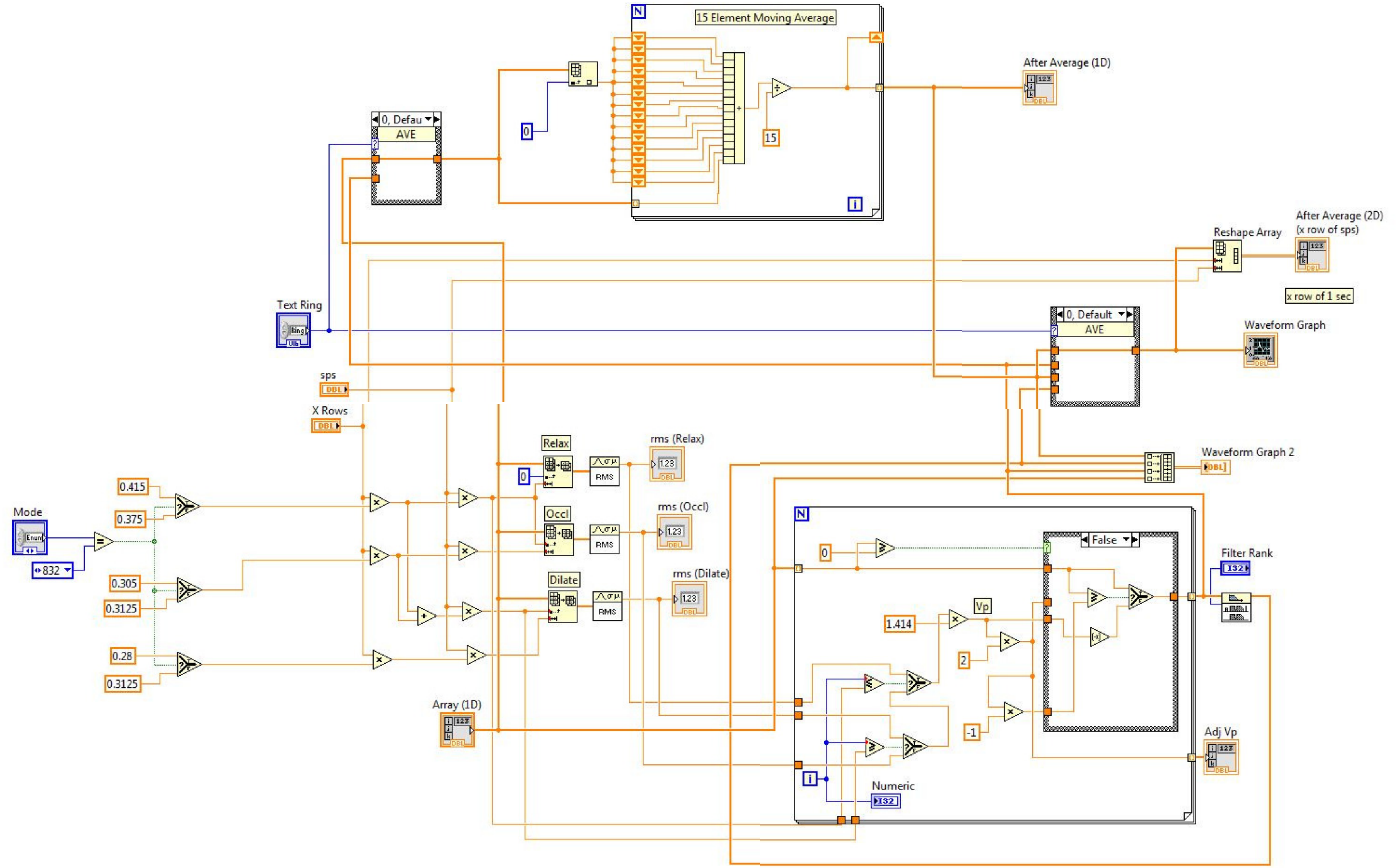

Supplementary Figure 5 Peak detection of waveform trend on occluded arm is done using LabVIEW Array Maximum and Minimum VI.

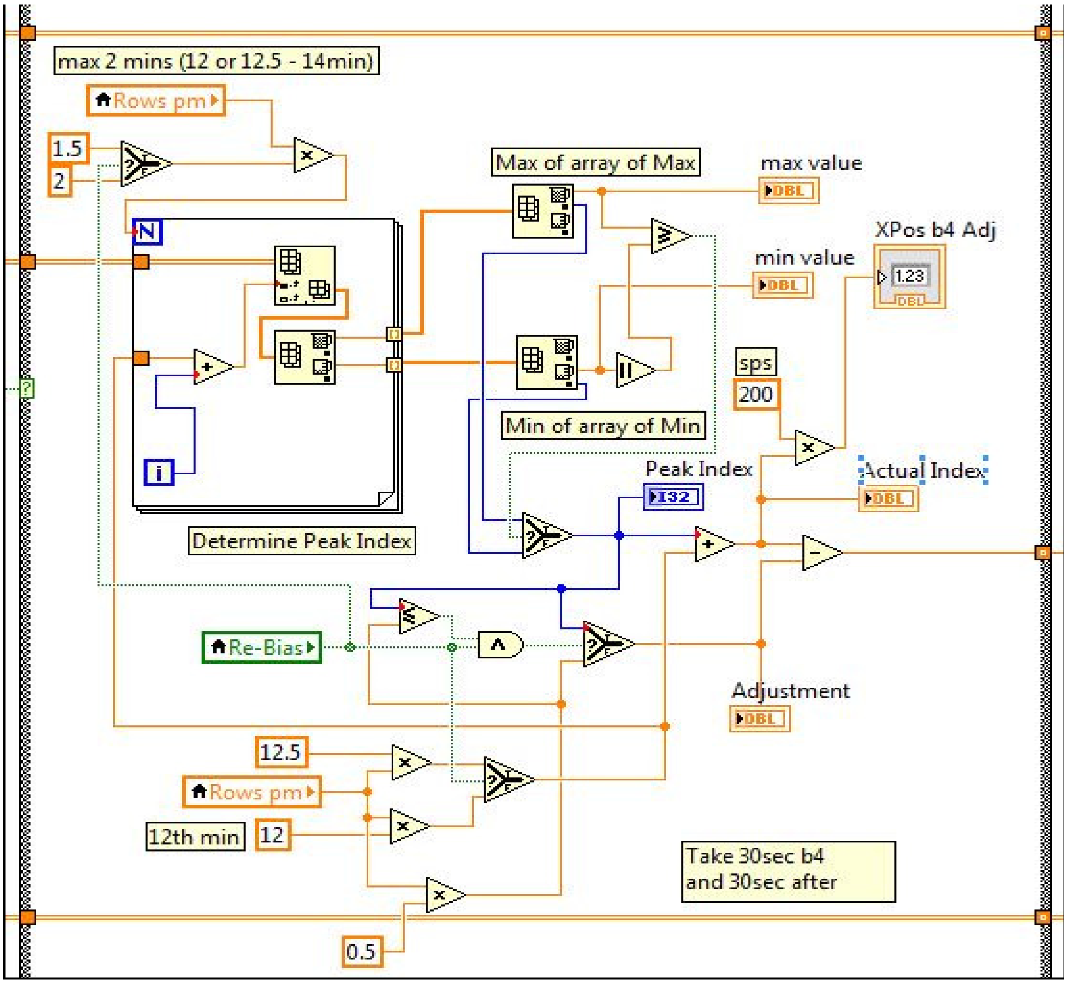

Supplementary Figure 6 Heart rate calculation by determination of peak per minute.

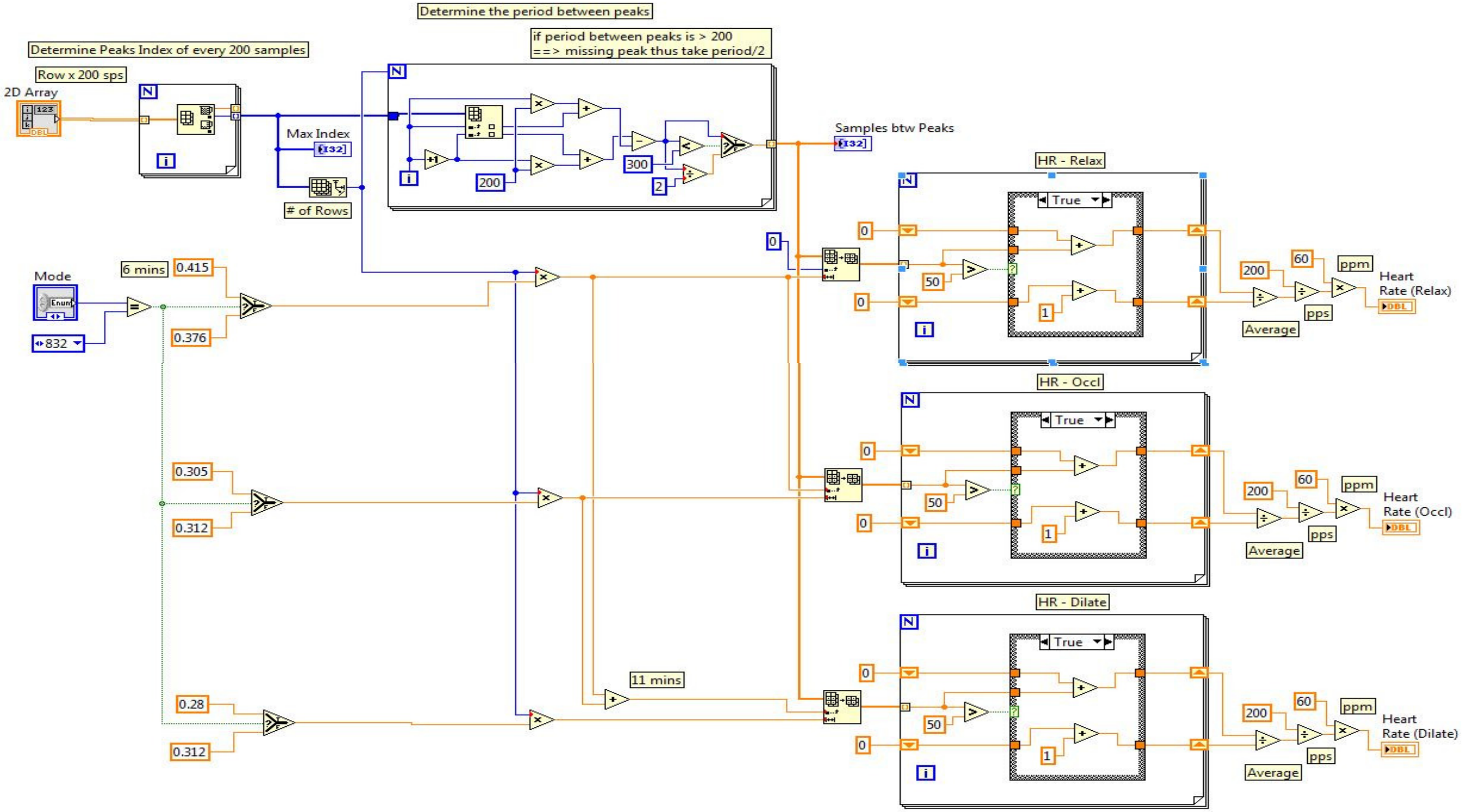

Supplementary Figure 7 Bland-Altman plot of the RA-MDI determined on 24 healthy volunteers on two separate occasions shows the mean difference to be within ±2 SD.

Mean Difference	SD	Mean Difference +2 SD	Mean Difference –2 SD
0.049840931	**0.140064512**	0.329969955	-0.230288093

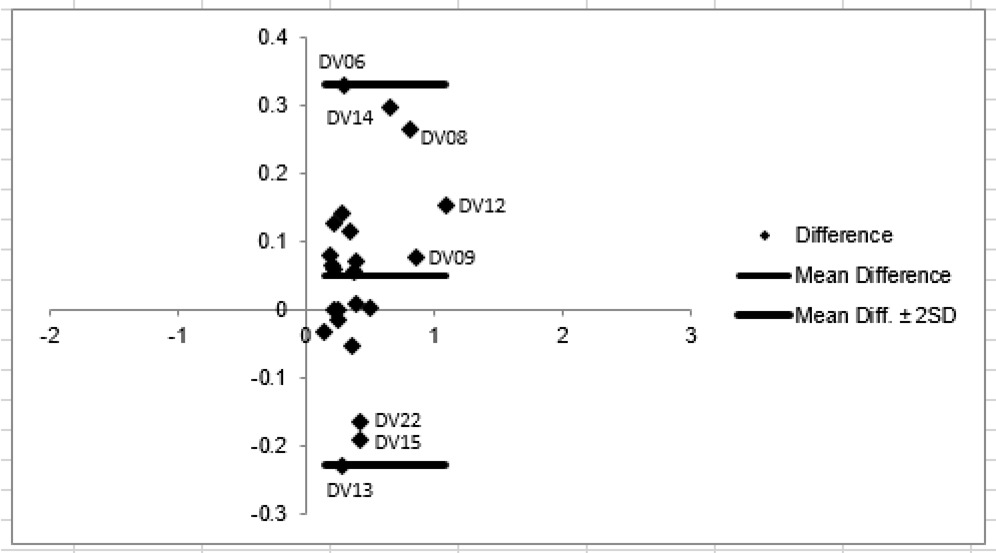

SD, significant deviation.

Supplementary Figure 8 Bland-Altman plot of the RA-MDI determined on 10 diabetes individuals on two separate occasions shows the mean difference to be within ±2 SD.

Mean Difference	SD	Mean Difference +2 SD	Mean Difference –2 SD
0.0595939	0.154273346	0.368140591	-0.248952791

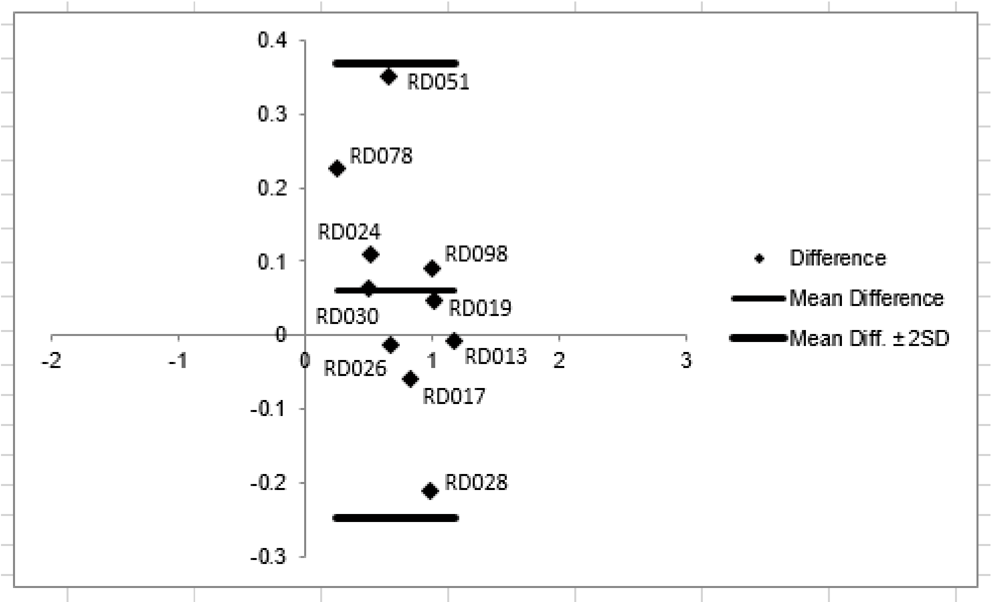

SD, significant deviation.
